# Flux-Free Diffusion Joining of SiC_p_/6063 Al Matrix Composites Using Liquid Gallium with Nano-Copper Particles in Atmosphere Environment

**DOI:** 10.3390/nano10030437

**Published:** 2020-02-29

**Authors:** Zeng Gao, Huanyu Yang, Jianguang Feng, Fei Ji, Jitai Niu, Josip Brnic

**Affiliations:** 1 School of Materials Science and Engineering, Henan Polytechnic University, Jiaozuo 454003, China; yanghuanyuyhy@163.com (H.Y.); jayfeng_cool@126.com (J.F.); jifei0108@163.com (F.J.); niujitai@163.com (J.N.); 2 School of Materials Science and Engineering, Harbin Institute of Technology, Harbin 150001, China; 3 Faculty of Engineering, University of Rijeka, 51000 Rijeka, Croatia; brnic@riteh.hr

**Keywords:** SiC_p_/Al matrix composites, diffusion joining, liquid gallium, nano-copper particles, microstructure

## Abstract

A new method for flux-free diffusion joining of aluminum matrix composites reinforced with SiC particles (SiC_p_/Al MMCs) in atmosphere environment has been developed. Liquid gallium and nano-copper particles were employed as filler metal under joining temperatures ranging between 400 °C to 480 °C, with a holding time of 2 h and pressure of 3 MPa. The results showed that 65 vol.% SiC_p_/6063 Al MMCs were successfully joined together. X-ray diffraction (XRD) analysis confirmed the presence of Ga_2_O_3_ at the fracture. Meanwhile, neither copper oxide nor aluminum oxide was detected. The formation of Ga_2_O_3_ can protect nano-copper particles and SiC_p_/6063 Al MMCs from oxidation. The width of weld seam tended to be narrowed from 40 μm to 14 μm gradually with increasing temperature from 400 °C to 480 °C. The maximum shear strength level of 41.2 MPa was achieved with a bonding temperature of 450 °C. The change of the strength was due to the adequate elements’ mutual diffusion and solution, as well as the change of the quantity and morphology of intermetallic compounds in the weld seam, such as Al_2_Cu and Cu_3_Ga. When the diffusion joining temperature reached 440 °C or above, the leak rate of the specimen remained under 10^−10^ Pa·m^3^/s.

## 1. Introduction

Aluminum matrix composites reinforced with SiC particles (SiC_p_/Al MMCs) represent advanced materials that possess unique mechanical and physical properties, such as high specific strength and specific modulus of elasticity, low thermal expansion coefficient, and high thermal conductivity [1,2,3,4,5,6,7]. Due to the uniform distribution of SiC particles in composites, components made from SiC_p_/Al MMCs are nearly isotropic and readily formable. Moreover, this material can be tailored to satisfy specific requirements via change of the volume ratio of SiC in composites, which makes it especially attractive for aerospace engineering, the automotive industry, and electronic packaging applications [8,9,10,11,12]. Joining is an important technological process to be used in these industrial applications involving SiC_p_/Al MMCs. However, the joining of aluminum matrix composites remains a key issue limiting the use of these advanced materials, due to the large difference between SiC and base aluminum in both physical and chemical properties.

The joining process has been investigated for SiC_p_/Al MMCs, mainly involving fusion welding, diffusion bonding, and brazing. In fusion welding methods, such as arc welding, the high temperature will deteriorate the matrix, the reinforcement particles, and the metal–ceramic interfacial bonds. Meanwhile, the high melting point of SiC will cause the molten pool to be viscous with poor fluidity, which is most likely to result in welding defects, such as blow holes, slag inclusions, SiC segregations, and incomplete backfill [13,14,15,16,17]. Diffusion bonding requires excessively high temperatures and pressures to preserve the age hardening treatment of the matrix and the quality of the ceramic–metal interface. In addition, the stable aluminum oxide film and the SiC particles on the surface of the composites form obstacles to diffusion and interfacial bonding, making it difficult to increase joint strength during diffusion bonding [18,19]. Most commercially available aluminum brazing materials involve temperatures above 500 °C, which degrade the age-hardened matrix. Additionally, it is hard to find a filler metal that can satisfactorily wet the base aluminum and SiC simultaneously. The current SiC_p_/Al MMCs brazing process has several disadvantages, such as work being performed under vacuum or controlled atmospheres, use of high temperature, and the use of polluting fluxes [20,21,22].

This work mainly describes a new method for flux-free diffusion joining of SiC_p_/Al MMCs in an atmosphere environment, using liquid gallium and nano-copper particles as filler metals. Gallium can form a low-temperature eutectic liquid phase with aluminum at about 27 °C [23]. At the same time, gallium possesses good solubility in aluminum of about 20wt.% at room temperature, as known from Al–Ga phase diagrams. Nano-copper particles have high surface energy, which is the main driving force of atomic motion that facilitates the annihilation of the vacancy cluster and bonding with SiC, as well as with aluminum in composites [24,25,26]. Different temperatures were considered in this study to evaluate the impact of nanoparticles on the microstructure evolution, as well as on the joint properties, such as shear strength and gas tightness.

## 2. Materials and Methods

**Figure 1:**
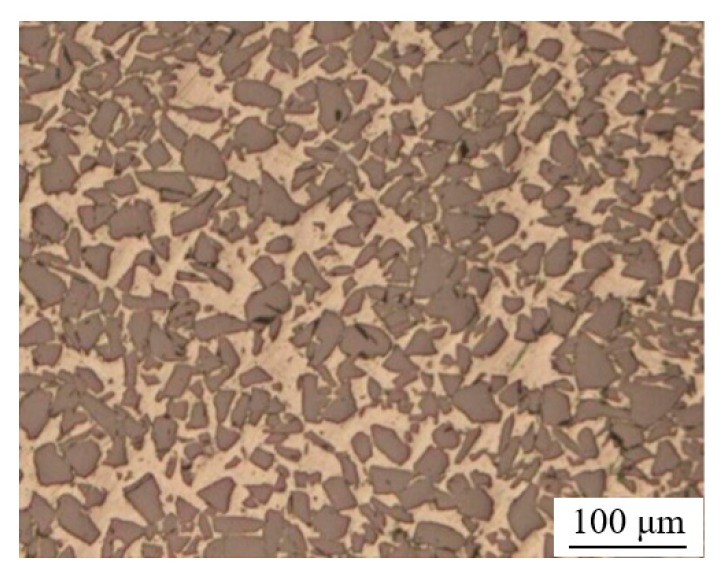
Metallographic structure of 65 vol.% aluminum matrix composites reinforced with SiC particles (SiC_p_/6063 Al MMCs).

The material to be bonded in this investigation was 6063 aluminum matrix composite reinforced with 65 vol.% SiC particles (65 vol.% SiC_p_/6063 Al MMCs). This material is extensively used for high precision structure servicing in a wide range of temperature conditions due to its low coefficient of thermal expansion (7.0 × 10^−6^/K). Uses for this material include electronic packaging modules, and structural components on satellites and space shuttles. The chemical composition of 6063 aluminum alloy is given in Table 1. The solid and liquid phase lines of 65 vol.% SiC_p_/6063 Al MMCs are in the range of 620–640 °C. The microstructure of as-received 65 vol.% SiC_p_/6063 Al MMCs is composed of uniformly distributed SiC particles and base aluminum, as presented in Figure 1. The purity of gallium used in this work was 99.99%, with a melting point of 29.7 °C. The nano-copper particles had a spherical appearance, with a diameter of around 50 nm. Meanwhile, the nano-copper particles were not oxidized and the purity was 99.9%.

**Figure 2:**
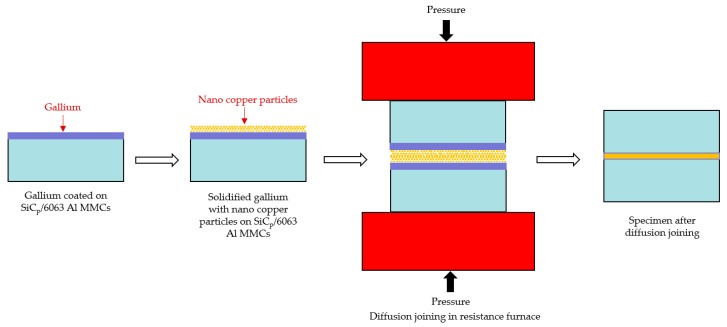
Schematic diagram of the flux-free diffusion joining of SiC_p_/6063 Al MMCs.

Specimens measuring 2.0 × 10.0 × 15.0 mm were machined from bulk material, which was manufactured by melt infiltration technology. Each half of a specimen was mechanically polished on the bonding side with 1200 grit SiC grinding paper, followed by rinsing in ethanol for 5 min. In order to improve the wettability of liquid gallium on composite surfaces, the samples were heated to 50 °C before and during the gallium deposition process. Then, a warmed (around 40–60 °C) soft cloth impregnated with an adequate amount of pure liquid gallium was utilized to smear a thin layer of liquid gallium on the bonded area. The thickness of gallium was kept at 1.7 μm (equal to 1 mg/cm^2^) by employing a high precision electronic scale. As soon as a layer of liquid gallium was formed on the SiC_p_/6063 Al MMCs surface, the sample was rapidly moved into a dry and low temperature environment to solidify the liquid gallium, aiming to minimize any possible reaction at room temperature between the liquid gallium and aluminum in the composites. After the previous process, the nano-copper particles were uniformly dropped onto the surface of gallium-treated SiC_p_/6063 Al MMCs. The mass ratio between gallium and copper was kept at 2:1. Then, the prepared samples of SiC_p_/6063 Al MMCs with gallium layers and nano-copper particles were brought into contact and were joined using preheated steel plates in a resistance furnace. Rapid heating of the specimens is one of the main requirements of this technology. Due to a certain amount of gallium that exists in the common interface, any delay in the heating stage will result in the intergranular diffusion of gallium, and thus a lack of liquid gallium at the interface. The resistance furnace used in this investigation was GWL-1200 and the temperature controlling accuracy was ±1°C. The stages of the flux-free diffusion joining in this research are shown schematically in Figure 2. The following diffusion joining temperatures were investigated: 400, 420, 440, 450, 460, and 480 °C, with a holding time of 2 h and pressure of 3 MPa.

**Figure 3:**
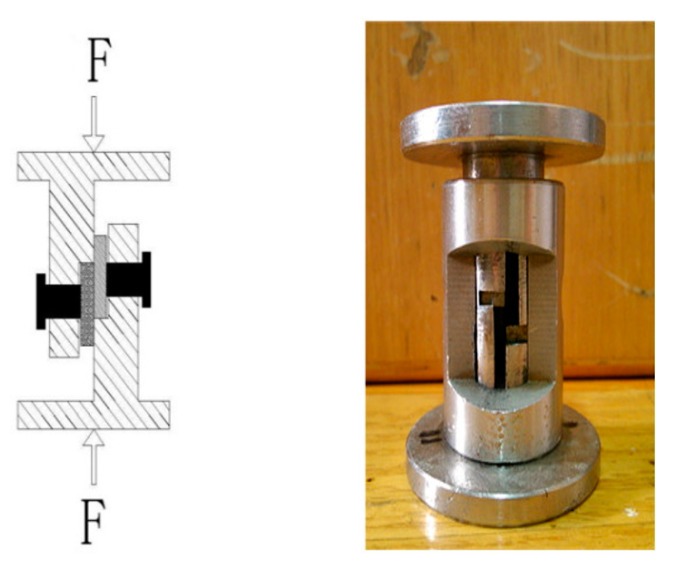
Schematic diagram of the shear test fixture and picture of the object.

Following diffusion joining, the shearing test was performed on the specimens at a constant rate of 0.2 mm/min and at room temperature by using an electronic universal testing machine (IIC-MST-100). A scheme of the instrument with the used sample for the shear test and a picture of the object are presented in Figure 3. Specimens were sectioned perpendicular to the bond interface and polished in a sequence of 400 to 1200 grit silicon carbide paper. Final surface preparation involved polishing with 0.25 μm diamond spray and clean water. Microscopic examination and fracture appearance of the joint were carried out by scanning electron microscope (FEI Quanta 200) coupled with energy dispersive X-ray spectroscopy (EDS), respectively. X-ray diffraction (XRD) was employed to analyze the phase in the joint. The joint gas tightness was measured with a ZQJ-530helium leak mass spectrometer.

## 3. Results and Discussion

### 3.1. Microstructure and Element Distribution Analysis of Diffusion Joints

**Figure 4:**
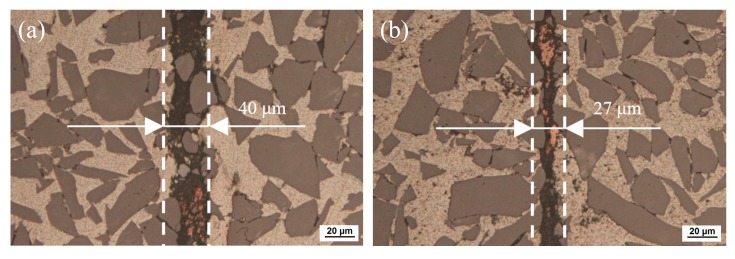

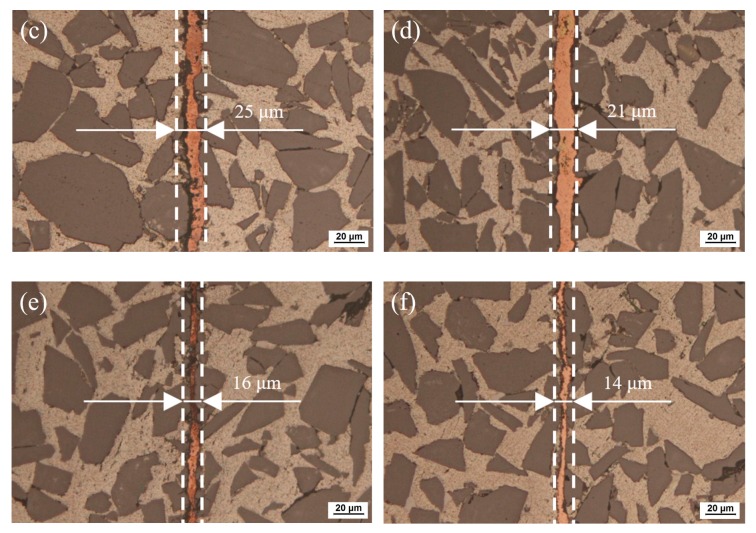
Optical micrographs of 65 vol.% SiC_p_/6063 Al MMC joint diffusion using liquid gallium with nano-copper particles at different temperatures: (**a**) 400 °C; (**b**) 420 °C; (**c**) 440 °C; (**d**) 450 °C; (**e**) 460 °C; (**f**) 480 °C.

The joining temperature has a great effect on the joint microstructure and properties. Figure 4 presents the joint microstructure evolution at different temperatures. Figure 4a,b show the appearance of a large number of SiC particles at the joint center. Theoretically, the presence of SiC particles in the weld center should be beneficial to joint properties due to the strengthening effect. However, the shearing and gas tightness tests in the following sections suggest that the properties of joints formed at 400 and 420 °C are unsatisfactory. This could be explained by several reasons. Lower joining temperatures, such as 400 °C and 420 °C, will cause the incomplete joining of nano-copper particles, and therefore some micro voids will inevitably be left in the weld seam during diffusion joining. In the subsequent cutting process of the metallographic specimen, the SiC particles on the cutting surface will be broken into small pieces and then embedded into the previous microvoids in the weld seam, as can be seen in Figure 4a,b. As a consequence, the joint properties, such as shearing strength and gas tightness, will be undesirable, which will be verified in the following sections. Although the melting point of pure copper is as high as 1083.4 °C, the nano-copper particles are diffusely coupled to one another and to SiC_p_/6063 Al MMCs at the joining temperature due to the nanometer effect. Nano-copper particles with a diameter of 50 nm have high surface energy, which is the main driving force of atomic motion that facilitates the annihilation of the vacancy cluster and diffusion into SiC_p_/6063 Al MMCs. It can be seen from Figure 4c to f that the weld seam is bright and continuous, where no nano-copper particles or SiC particles can be found. Meanwhile, the width of the weld seam tends to narrow gradually with temperature increase. As shown in Figure 4a to f, the width of weld seam is about 40 μm when joined at 400 °C, while this value is 14 μm when joined at 480 °C. The weld seam formed at 450 °C is quite even and uniform. With increase of the diffusion joining temperature, the compactness of nano-copper particles will increase in the joining process as well. In addition, the interaction between SiC_p_/6063 Al MMCs and liquid gallium with nano-copper will increase with increasing joining temperature. Both of those reasons will result in the decrease of the weld seam width.

**Figure 5:**
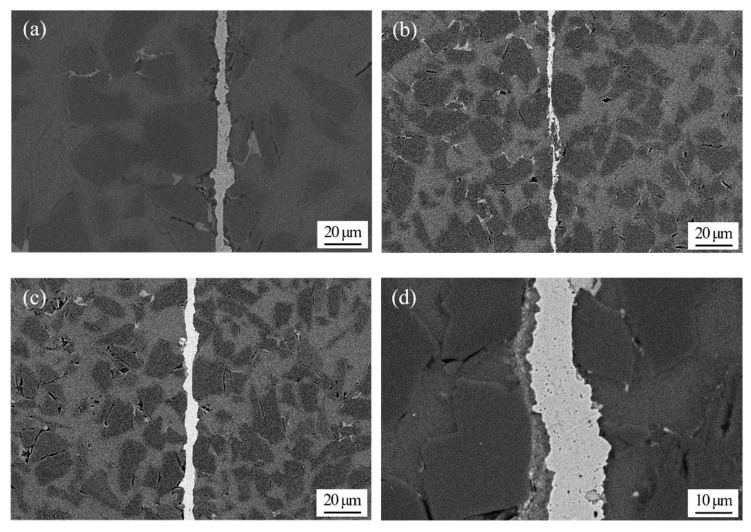
Scanning electron microscope (SEM) micrographs of the joint made using liquid gallium with nano-copper particles at: (**a**) 460 °C; (**b**) 480 °C; and (**c**,**d**) 450 °C.

Figure 5 shows the scanning electron microscope (SEM) micrographs of joints made at temperatures of 450 °C, 460 °C, and 480 °C. Due to the higher diffusion joining temperatures, the weld seams are free from SiC particles and nano-copper particles, as can be seen in Figure 5. Meanwhile, Figure 5 also illustrates that the bonds between copper and SiC and between copper and parent aluminum are very close, and no void can be found at the interface regions. This would be beneficial for joint mechanical properties and gas tightness properties. Figure 5d shows that the joint made at 450 °C consists of bilayer structure, due to the interaction between nano-copper, liquid gallium, and SiC_p_/6063 Al MMCs.

**Figure 6:**
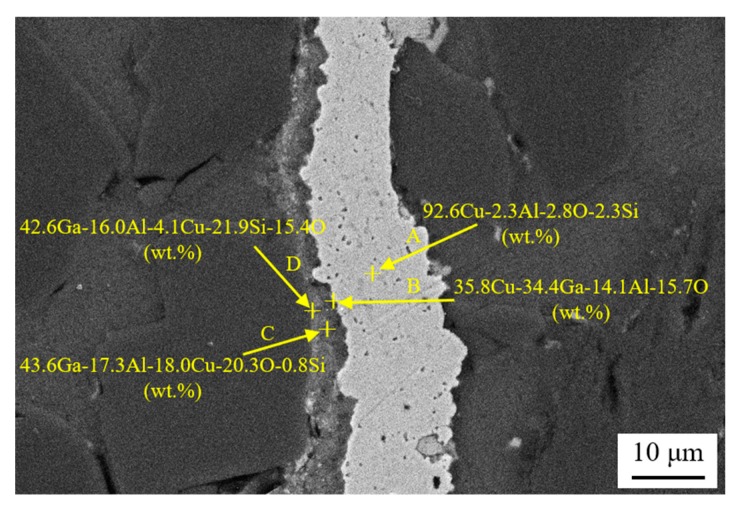
Energy dispersive X-ray spectroscopy (EDS) analysis of the joint made using liquid gallium with nano-copper particles at 450 °C.

The chemical composition of the bilayer structure was analyzed using EDS, as shown in Figure 6. Testing points A, B, C, and D were located at the center of the first layer, the interface between the first and second layers, the center of the second layer, and the interface between the second layer and SiC_p_/6063 Al MMCs, respectively. At point A, the chemical composition was measured as 92.6Cu-2.3Al-2.8O-2.3Si (wt.%). Obviously, Cu was the primary composite in this white region. Points B and C mainly consisted of Cu, Ga, Al, and O, and these elements can form a solid solution or intermetallic compound. Testing results suggested that the Cu concentration decreased gradually from the joint center to the interface. Meanwhile, the Ga and Al concentration increased gradually. Oxidation was inevitable, since the diffusion joining was carried out in an atmosphere environment and no flux was utilized. Figure 6 revealed that the joint contained some O, which was mainly located at the second layer. The concentration of O in that region was between 15.4 wt.% to 20.3 wt.%. In the first layer, a very small amount of O was found, the value of which was determined to be 2.8 wt.%. Therefore, it can be concluded that the nano-copper particles in the weld seam were almost not oxidized due to the protection provided by liquid gallium at the interface.

**Figure 7:**
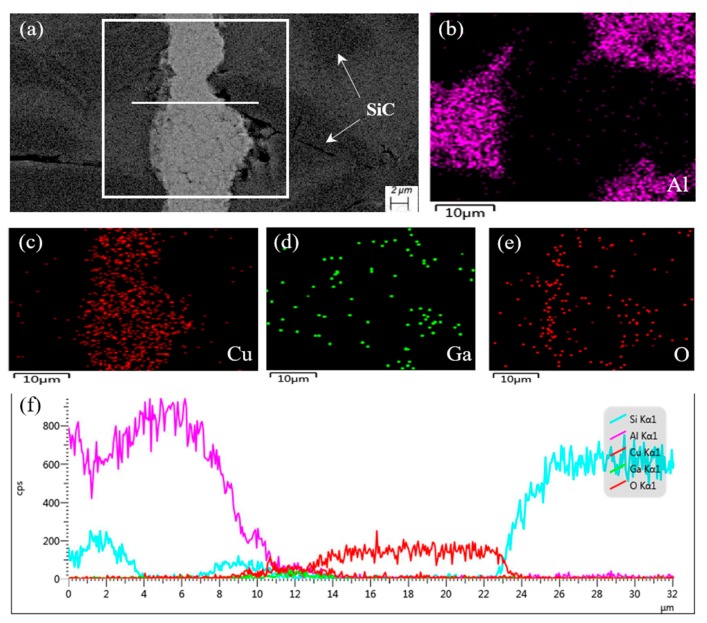
SEM image of typical region at the interface of the 65 vol.% SiC_p_/6063 Al MMC joint made at 450 °C and corresponding energy dispersive X-ray maps showing distribution of elements: (**a**) SEM image; (**b**–**e**) individual elemental mapping of Al, Cu, Ga, and O, respectively; (**f**) line scanning profile.

Figure 7 shows SEM images of the joint and the corresponding elemental mapping, as well as the line scanning profile. Figure 7b,c shows the interaction between Al and Cu near the joint. Due to the high activity of nano-copper particles in the joint, Cu will diffuse into the composites along the grain boundary, as well as at the interfaces between SiC particles and base aluminum, forming intermetallic compounds (mainly CuAl_2_) or an Al-based solid solution. As can be seen in Figure 7d, Ga distributes uniformly in the whole scanning region, except in the SiC particle regions. Meanwhile, the O has a similar distribution as the Ga, as shown in Figure 7e. This may indicate that the oxygen is fixed by gallium during diffusion joining. A possible reaction mechanism is described in Equation (1).

2Ga + 3O → Ga_2_O_3_, (1)

The formation of Ga_2_O_3_ can protect nano-copper particles and the surfaces of SiC_p_/6063 Al matrix composites from oxidation. Meanwhile, intermetallic compounds (mainly Cu_3_Ga) or Cu-based solid solution will be formed between the Cu and Ga elements, according to the Cu–Ga phase diagram. Figure 7f shows the element distribution, along with the scanning line indicated in Figure 7a. It can be seen that at the interface of joint (near the 12 μm location) that the intensity of Cu and Ga increases gradually, which is mainly because of the formation of Al_2_Cu and GaO_3_ in this area.

### 3.2. Mechanical Properties of Diffusion Joints

**Figure 8:**
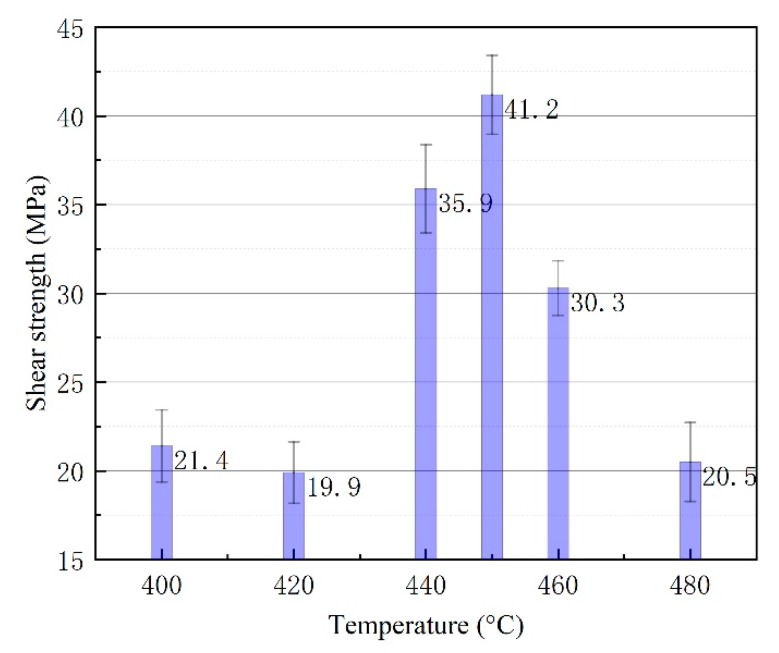
Shear strength of diffusion joints made at different temperatures.

The pretreated samples of SiC_p_/6063 Al MMCs with gallium layers and nano-copper particles were diffusion joined according to the process in Section 2 (2 h at 400–480 °C with 3 MPa pressure in air). Shear tests were then performed to determine the diffusion joint fracture resistance. The diffusion coefficient of the filler metal varied directly with the diffusion joining temperature. With temperature increase, the diffusion coefficient of the elements increased significantly, which had significant effects on joint shear strength. Figure 8 shows the shear strength of the diffusion joints at different temperatures. Five specimens were tested for each joining condition. The maximum shear strength level of 41.2 MPa was achieved for the bonding temperature of 450 °C. The error between average and measured values was below 3.1 at the joining temperature of 450 °C. When the bonding temperature decreased to 440 °C, 420°C, and 400 °C, the shear strength decreased to 35.9 MPa, 19.9 MPa, and 21.4 MPa, respectively. The corresponding error values at those temperatures were below 3.6. Further increases in bonding temperature to 460 °C and 480 °C similarly caused a reduction of the shear strength to 30.3 MPa and 20.5 MPa. The error values at the joining temperatures of 460 °C and 480 °C were below 1.9 and 2.7, respectively. The change of the strength was due to the adequate mutual diffusion of elements and the solution on the one hand, and on the other hand was due to the change of the quantity and morphology of the intermetallic compound (such as Al_2_Cu and Cu_3_Ga) phase in the weld seam having a remarkable influence on the shear strength as the joining temperature increased.

### 3.3. Fracture Analysis of Diffusion Joints

**Figure 9:**
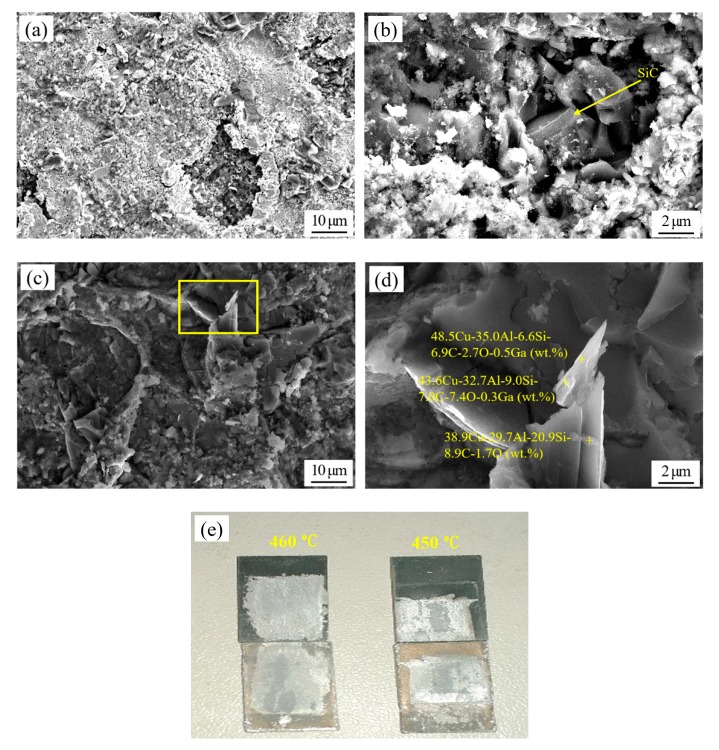
Scanning fracture appearance and EDS analyses of the joints made using liquid gallium with nano-copper particles at (**a**,**b**) 450 °C and (**c**,**d**) 460 °C. (**e**) Cross-section of joints after shear test.

**Figure 10:**
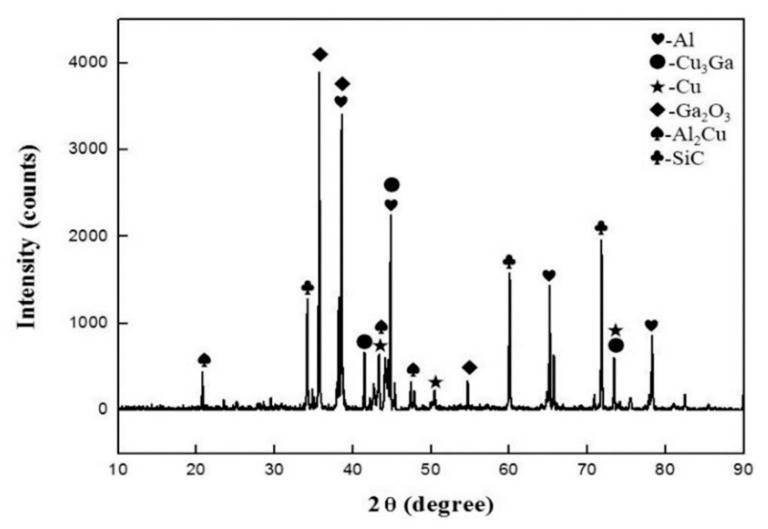
X-ray diffraction XRD analysis of the joint fracture made using liquid gallium with nano-copper particles at 450 °C.

For the SiC_p_/6063 Al-MMCs with different diffusion joining processes, the joint fracture can present diversiform appearances, and the effect of temperature on the fracture is mainly discussed in this research. As can be seen in Figure 9a,b, the fracture is generally in the form of a quasi-cleavage fracture. Additionally, the fracture primarily occurs in the filler metal, and a part of the fracture occurs at the interface between the filler metal and SiC_p_/6063 Al-MMCs, since the SiC particle can be seen in the fracture. The fracture also demonstrates that there is no large blocked intermetallic compound in the joint, although a small intermetallic compound is found in the fracture, as seen in the latter XRD analysis. This would be very beneficial in terms of increasing the joint strength [27]. When the diffusion joining temperature increases to 460 °C, the fracture appearance changes significantly, as shown in Figure 9c,d. Cleavage fracture is the main fracture mechanism for the joint made at 460 °C. The fracture occurs in the filler metal, and no SiC particle can be found at the fracture surface. A large number of brittle intermetallic compounds formed during the diffusion joining, due to the high activity of nano-copper particles and the nature of the binary Al–Cu compound. As can be seen in Figure 9d, the EDS analysis revealed that a mass of Al and Cu elements exist in the fracture. The atomic ratio of Al and Cu in the analyzed region is quite close to the intermetallic compound Al_2_Cu. Therefore, the presence of large amounts of brittle Al_2_Cu is the main reason for the decreasing joint shear strength. The formation of brittle Al_2_Cu in the filler metal initiates cracks in the joint [28]. The cross-sections of the joints made at 450 °C and 460 °C after shear tests are displayed in Figure 9e. XRD analysis of the fracture surface is presented in Figure 10 and reveals peaks for the intermetallic compounds Al_2_Cu and Cu_3_Ga when the bonding temperature of 450 °C was applied. Meanwhile, some Ga_2_O_3_ was found at the fracture surface, while copper and aluminum oxides were not detected. This indicates that the oxide on 65 vol.% SiC_p_/6063 Al MMCs surface can be removed thoroughly via the strong permeability of gallium, and that the gallium can also protect the nano-copper particles and base aluminum from oxidation damage during diffusion joining in an atmosphere environment.

### 3.4. Gas Tightness of Joints Produced at Different Diffusion Temperatures

**Figure 11:**
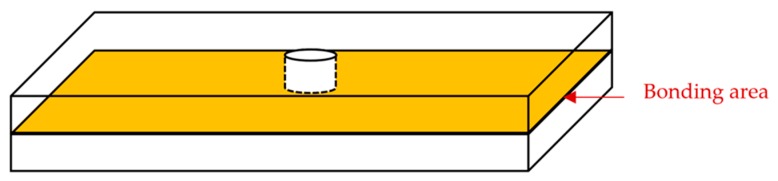
Schematic diagram of specimen for gas tightness test.

One of the important application areas of high volume SiC_p_/6063 Al MMCs is in electronic packaging, which requires a very strict gas tightness to protect the inside chip from potential damage caused by surrounding air. After diffusion joining, the bonding area may become the only potential leakage path if bonding defects exist, such as micro-cracks, gaps, porosity, and lack of bonding. In order to test the gas tightness of the joint, a specimen with a hole in the bonding surface in the center area was prepared and bonded, as shown in Figure 11.

As a qualified component, the gas leakage rate after diffusion joining is supposed to remain at 10^−8^ Pa·m^3^/s or below. After diffusion joining, gas tightness was measured with a ZQJ-530 helium leak mass spectrometer. Testing of a specimen was done by putting it on top of a soft vinyl plate containing a hole leading to a vacuum hose. Vacuum strength of 10^−10^ Pa was applied from underneath to the inside of the hole in the specimen. Helium gas was then sprayed on top of the specimen and helium leakage through the joint and base material was measured. A vacuum drop of 4 decades indicated that the sample was not gas tight. The testing results are displayed in Table 2. It is apparent that the leakage rate after diffusion joining meets the requirement once the bonding temperature reaches 440 °C or higher. When the bonding temperature is 400 °C or 420 °C, the leakage rate after diffusion joining is 10^−6^ Pa·m^3^/s, which is too high and fails to meet the requirement for packaging components. Under diffusion pressure of 3 MPa and holding time of 2 h, the lower joining temperatures, such as 400 °C and 420 °C, cause incomplete joining of nano-copper particles, reducing the compactness of the weld seam due to microvoids inevitably being left in the weld seam. That can be seen from the optical micrographs in Figure 4a,b. Hence, the gas tightness of the specimens joined at temperatures of 400 °C and 420 °C is undesirable. The stability tests were carried out after one week with the same method. As shown in Table 2, the leakage rate of the specimens remains unchanged.

Table 2 suggests that the diffusion temperature plays a very important role in the leakage rate of specimens. This can be explained by the diffusion behavior between filler materials and SiC_p_/6063 Al MMCs. The diffusion coefficient increases remarkably with the increasing joining temperature. With large diffusion coefficient, the nano-copper particles in the weld seam are sintered to ensure they are more complete. Meanwhile, the interfaces between filler materials and SiC_p_/6063 Al MMCs are more compact. Hence, it can be found that the leakage rate of specimens produced at temperatures of 440 °C and above is quite low, reaching 10^−10^ Pa·m^3^/s.

## 4. Conclusions

In this work, 65 vol.% SiC_p_/6063 Al MMCs were successfully joined through diffusion joining in an atmosphere environment. Liquid gallium and nano-copper particles were employed as filler metals at joining temperatures of 400-480 °C, with a holding time of 2 hours and pressure of 3 MPa. After diffusion joining, microstructure, element distribution, and fracture analyses, as well as shear testing and gas tightness testing of the joints, were carried out to understand the joint quality. The observations are summarized as follows:

(1) Optical microscopy showed that lower joining temperatures of 400 °C and 420 °C will cause incomplete joining of nano-copper particles. Once the joining temperature reached 440 °C or above, the nano-copper particles were diffusion-joined together with each other and with SiC_p_/6063 Al MMCs due to the nanometer effect. The width of the weld seam tended to narrow from 40 μm to 14 μm gradually with the temperature increase from 400 °C to 480 °C. The formation of Ga_2_O_3_ can protect nano-copper particles and the surfaces of SiC_p_/6063 Al matrix composites from oxidation.

(2) The maximum shear strength level of 41.2 MPa was achieved when a bonding temperature of 450 °C was utilized. Lower of higher bonding temperatures will cause a reduction of the shear strength. The change of the strength lies in the adequate elements’ mutual diffusion and the solution on the one hand, and on the other hand was due to the change of the quantity and morphology of the intermetallic compound (such as Al_2_Cu and Cu_3_Ga) phase in the weld seam having a remarkable influence on the shear strength as the joining temperature increased.

(3) At the optimal diffusion temperature, the joint fracture was generally in the form of a quasi-cleavage fracture. The fracture primarily occurred in the filler metal, and a part of the fracture occurred at the interface between the filler metal and SiC_p_/6063 Al-MMCs. Cleavage fracture was the main fracture mechanism for the joints made at 460 °C or higher, caused by the formation of a large number of brittle intermetallic compounds, such as Al_2_Cu. XRD analysis revealed the formation of Al_2_Cu and Cu_3_Ga when a bonding temperature of 450 °C was applied. Ga_2_O_3_ was found on the fracture surface, while copper and aluminum oxides were not detected.

(4) The leakage rate of specimens reduced with increasing diffusion joining temperature. When the diffusion temperature reached 440 °C or above, the leakage rate of the specimens remained under 10-10 Pa·m^3^/s, which met the requirements for packaging components. The leakage rate of the specimens remained unchanged after one week, exhibiting satisfactory stability.

## Figures and Tables

**Table 1:** Chemical composition of 6063 aluminum alloy (in wt.%).

Element	Si	Mg	Fe	Cu	Mn	Cr	Zn	Ti	Al
wt.%	0.20–0.60	0.45–0.90	0.35	0.10	0.10	0.10	0.10	0.10	Balance

**Table 2:** Gas tightness of diffusion joints bonded by liquid gallium with nano-copper particles at different temperatures.

Temperature (°C)	400	420	440	450	460	480
**Leak rate after diffusion joining (Pa·m^3^/s)**	10^−6^	10^−6^	10^−10^	10^−10^	10^−10^	10^−10^
**Leak rate after one week (Pa·m^3^/s)**	10^−6^	10^−6^	10^−10^	10^−10^	10^−10^	10^−10^
